# miRNA-Coordinated Schizophrenia Risk Network Cross-Talk With Cardiovascular Repair and Opposed Gliomagenesis

**DOI:** 10.3389/fgene.2020.00149

**Published:** 2020-03-04

**Authors:** Hongbao Cao, Ancha Baranova, Weihua Yue, Hao Yu, Zufu Zhu, Fuquan Zhang, Dongbai Liu

**Affiliations:** ^1^ Department of Psychiatry, First Hospital/First Clinical Medical College of Shanxi Medical University, Taiyuan, China; ^2^ Department of Genomics Research, R&D Solutions, Elsevier Inc., Rockville, MD, United States; ^3^ School of Systems Biology, George Mason University (GMU), Fairfax, VA, United States; ^4^ Research Center for Medical Genetics, Moscow, Russia; ^5^ Department of Psychiatry Institute of Mental Health, Peking University, Bejing, China; ^6^ Department of Psychiatry, Jining Medical University, Jining, China; ^7^ Department of Neurology, Jiangyin People's Hospital Affiliated to Southeast University, Jiangyin, China; ^8^ Department of Psychiatry, The Affiliated Brain Hospital of Nanjing Medical University, Nanjing, China

**Keywords:** miRNA, schizophrenia, miR-208b-3p, miR-208a-3p, miR-494-5p, gliomagenesis, quaking, heart disease

## Abstract

**Background:**

Schizophrenia risk genes are widely investigated, but a systemic analysis of miRNAs contributing to schizophrenia is lacking.

**Methods:**

Schizophrenia-associated genetic loci profiles were derived from a genome-wide association study (GWAS) from the Schizophrenia Working Group of the Psychiatric Genomics Consortium (PGC) dataset. Experimentally confirmed relationships between miRNAs and their target genes were retrieved from a miRTarBase. A competitive gene set association analysis for miRNA-target regulations was conducted by the Multi-marker Analysis of GenoMic Annotation (MAGMA) and further validated by literature-based functional pathway analysis using Pathway Studio. The association between the targets of three miRNAs and schizophrenia was further validated using a GWAS of antipsychotic treatment responses.

**Results:**

Three novel schizophrenia-risk miRNAs, namely, miR-208b-3p, miR-208a-3p, and miR-494-5p, and their targetomes converged on calcium voltage-gated channel subunit alpha1 C (CACNA1C) and B-cell lymphoma 2 (BCL2), and these are well-known contributors to schizophrenia. Both miR-208a-3p and miR-208b-3p reduced the expression of the RNA-binding protein Quaking (*QKI*), whose suppression commonly contributes to demyelination of the neurons and to ischemia/reperfusion injury. On the other hand, both QKI and hsa-miR-494-5p were involved in gliomagenesis.

**Conclusion:**

Presented results point at an orchestrating role of miRNAs in the pathophysiology of schizophrenia. The sharing of regulatory networks between schizophrenia and other pathologies may explain higher cardiovascular mortality and lower odds of glioma previously reported in psychiatric patients.

## Introduction

Schizophrenia, a common psychiatric condition characterized by abnormal social behavior and failure to understand reality, affects up to 1% of the human population and causes substantial morbidity and mortality (Barnett, 2018). It is a complex disorder with an estimated heritability of around 80% and an unclear mode of genetic transmission (Hilker et al., 2018). There are many risk genes for schizophrenia, and there is a very small risk attributed to each one (Winchester et al., 2014; Li et al., 2016). In the largest multi-stage genome-wide schizophrenia association study to date, with 34,241 cases, 45,604 controls, and 1,235 affected parent–offspring trios, a total of 128 independent associations spanning 108 conservative loci were identified (Ripke et al., 2014), many of which were consistent with leading pathophysiological hypotheses of schizophrenia development.

It is worth noting that schizophrenia rarely results from the disruption of an individual gene, or even a contiguous chromosomal region. On the contrary, this condition is commonly attributed to the concerted and stable dysregulation of a complex genetic network or a set of networks (Gilman et al., 2012). Because of that, the dysregulation of master regulatory molecules, such as miRNAs, is expected to play a crucial role in the pathogenesis of schizophrenia. Indeed, altered levels of miRNA in the brain, in peripheral blood mononuclear cells, and in serum are found in patients with schizophrenia (Xu et al., 2010; Wang et al., 2014). Consequently, miRNAs that systemically regulate the genes contributing to the risk of schizophrenia may be of particular importance to its pathophysiology.

In this study, we investigated the miRNA-target gene set associated with schizophrenia with a goal of pinpointing potential master miRNA regulators of the gene networks associated with this disorder. To do that, we selected all experimentally confirmed miRNA–target interactions (MTIs) previously collected in a manually curated miRTarBase (Huang et al., 2020), and we then linked them to a schizophrenia-related tissue context through performing a MAGMA analysis (de Leeuw et al., 2015) of confirmed genes rather than miRNAs itself. The finding was validated by PPI network building and an analysis of secondary GWAS datasets concerning differential antipsychotic treatment responses. Our study prioritizes three miRNAs, miR-208a, miR-208b, and miR-494, as potential high-level regulators of schizophrenia phenotypes.

## Methods and Materials

### Experimentally Confirmed Pairs of miRNA With Their Target Genes

In order to get the most reliable connection, only miRNA–target pairs supported by strong experimental evidence (reporter assay or Western blot) were retrieved from miRTarBase 7.0. (http://mirtarbase.mbc.nctu.edu.tw/php/download.php) (Chou et al., 2017).

### Competitive Gene Set Association and Literature-Based Pathway Analysis

A Multi-marker Analysis of GenoMic Annotation (MAGMA) based on a multiple linear principal components regression model was previously designed to analyze the gene set association involved in genome-wide association studies (GWAS) data (de Leeuw et al., 2015). For each miRNA, its target genes were treated as a gene set, and then the competitive MAGMA-based gene-set analysis was utilized to test the association of each gene set using the summary statistics from the PGC2 GWAS (Ripke et al., 2014). The European samples from the 1,000 Genomes data (http://www.1000genomes.org) were used as reference data sets for the summary statistics gene analysis. Potentially confounding effects of gene size and gene density were treated as covariates in a generalized regression model. Multiple comparisons were corrected by a threshold of the false discovery rate (FDR) < 0.05. Then, significantly associated miRNA target sets were validated using the summary result of a GWAS of antipsychotic treatment responses in 2,413 schizophrenia patients (Yu et al., 2018). East Asian samples from the 1,000 Genomes data (http://www.1000genomes.org) were used as reference data sets for the summary statistics gene analysis.

The literature-based pathway analysis has been conducted using Pathway Studio (www.pathwaystudio.com), which allowed us to explore potential functional connections of miRNAs, their targets and schizophrenia by providing high-quality coverage of these connections with evidence extracted from full-text scientific reports.

## Results

### miRNA–Target Gene Regulating Relationships

A total of 8,496 unique miRNA–target pairs were retrieved from miRTarBase, involving 740 miRNAs and 2,853 target genes (**Supplementary Table S1**). After exclusion of all miRNAs with only one target gene each, a total of 539 miRNAs with two or more targets each were subjected to a gene set association analysis. For each miRNA, its target genes formed a gene set (N = 539). Taken together, all gene sets were comprised of 2,726 unique genes defined in PGC2 genotype data (**Supplementary Table S2**). The statistics describing miRNA–target gene regulations are shown in **Table 1**.

**Table 1:** miRNA–target gene sets associated with schizophrenia.

miRNA	nGenes	Beta	S.E.	*p*	FDR
miR-208b-3p	4	3.8	0.607	2.04E-10	1.10E-07
miR-494-5p	3	2.53	0.708	1.72E-04	0.031
miR-208a-3p	8	1.43	0.4	1.75E-04	0.031
miR-146b-5p	17	0.886	0.275	6.52E-04	0.088
miR-599	2	2.73	0.881	9.81E-04	0.106
miR-4782-3p	3	2.13	0.812	4.40E-03	0.364
miR-466	2	2.68	1.05	5.37E-03	0.364
miR-21-5p	131	0.233	0.092	5.89E-03	0.364
miR-126-3p	43	0.389	0.155	6.08E-03	0.364
miR-29c-5p	4	1.32	0.541	7.30E-03	0.369
miR-33a-5p	31	0.425	0.179	8.68E-03	0.369
miR-153-5p	4	1.41	0.592	8.70E-03	0.369
miR-10a-5p	20	0.574	0.242	8.90E-03	0.369

nGenes: the number of target genes for the miRNA; Beta, the regression coefficient for target gene set analysis; S.E., the standard error of the regression coefficient; FDR, the false discovery rate.

### Novel miRNAs Contributing to the Risk of Schizophrenia

Competitive gene set association analysis conducted by MAGMA identified three miRNAs as significantly associated with schizophrenia, namely, hsa-miR-208b-3p (miR-208b) (*p* = 2.04E-10, FDR = 1.10E-7), hsa-miR-494-5p (miR-494) (*p* = 1.72E-4, FDR = 0.031), and hsa-miR-208a-3p (miR-208a) (p = 1.75E-4, FDR = 0.031). An analysis of expression for these miRNAs was performed in a comprehensive miRmine dataset (Panwar et al., 2017) that was comprised of 304 high-quality microRNA sequencing experiments. Two of the three miRNAs studied, namely, miR-208b-3p and miR-494-5p, were expressed in various brain tissues at substantial levels. Notably, the expression pattern of miR-208b-3p was restricted to brain and plasma, while miRNA miR-208a-3p was specific to serum, plasma, and placenta. While neither heart nor muscle has been covered by the miRmine dataset, a body of work has demonstrated the importance of miR-208a-3p and miR-208b-3p as myoMiRs, expressed in heart tissues along with their myosin heavy chain encoding genes MYH6 and MYH7, respectively (Siddique et al., 2016).

**Table 2** presents a list of target genes regulated by these three miRNAs. Interestingly, all three highlighted miRNAs directly target CDKN1A, pointing to its possible function as a hub gene in the pathology of schizophrenia.

**Table 2:** Experimentally confirmed target genes of the three miRNAs contributing to the risk of schizophrenia.

miRNA	Target	Experiments	PMID
miR-208a-3p	CACNA1C	Luciferase reporter assay	27545043
miR-208a-3p	CACNB2	Luciferase reporter assay	27545043
miR-208a-3p	CDKN1A	qRT-PCR//Luciferase reporter assay//Western blot	20190813
miR-208a-3p	CDKN1A	Luciferase reporter assay//Western blot	26754670
miR-208a-3p	ETS1	Luciferase reporter assay//Microarray//qRT-PCR//Western blot	20576608
miR-208a-3p	MED13	Luciferase reporter assay//qRT-PCR//Western blot	17379774
miR-208a-3p	PDCD4	Luciferase reporter assay//qRT-PCR//Western blot	27634902
miR-208a-3p	QKI	Luciferase reporter assay	28283792
miR-208a-3p	SOX6	Luciferase reporter assay//Western blot	25023649
miR-208b-3p	CACNA1C	Luciferase reporter assay	27545043
miR-208b-3p	CACNB2	Luciferase reporter assay	27545043
miR-208b-3p	CDKN1A	qRT-PCR//Luciferase reporter assay//Western blot	20190813
miR-208b-3p	CDKN1A	Luciferase reporter assay//Microarray//qRT-PCR//Western blot	26044724
miR-208b-3p	QKI	Luciferase reporter assay	28283792
miR-494-5p	CXCR4	Luciferase reporter assay//qRT-PCR//Western blot	25955111
miR-494-5p	DPYD	GFP reporter assay//qRT-PCR//Western blot	25873402
miR-494-5p	PTEN	Luciferase reporter assay//qRT-PCR//Western blot	26045065

### Multiple Functional Pathways Link the Three miRNAs to Schizophrenia

**Figure 1:**
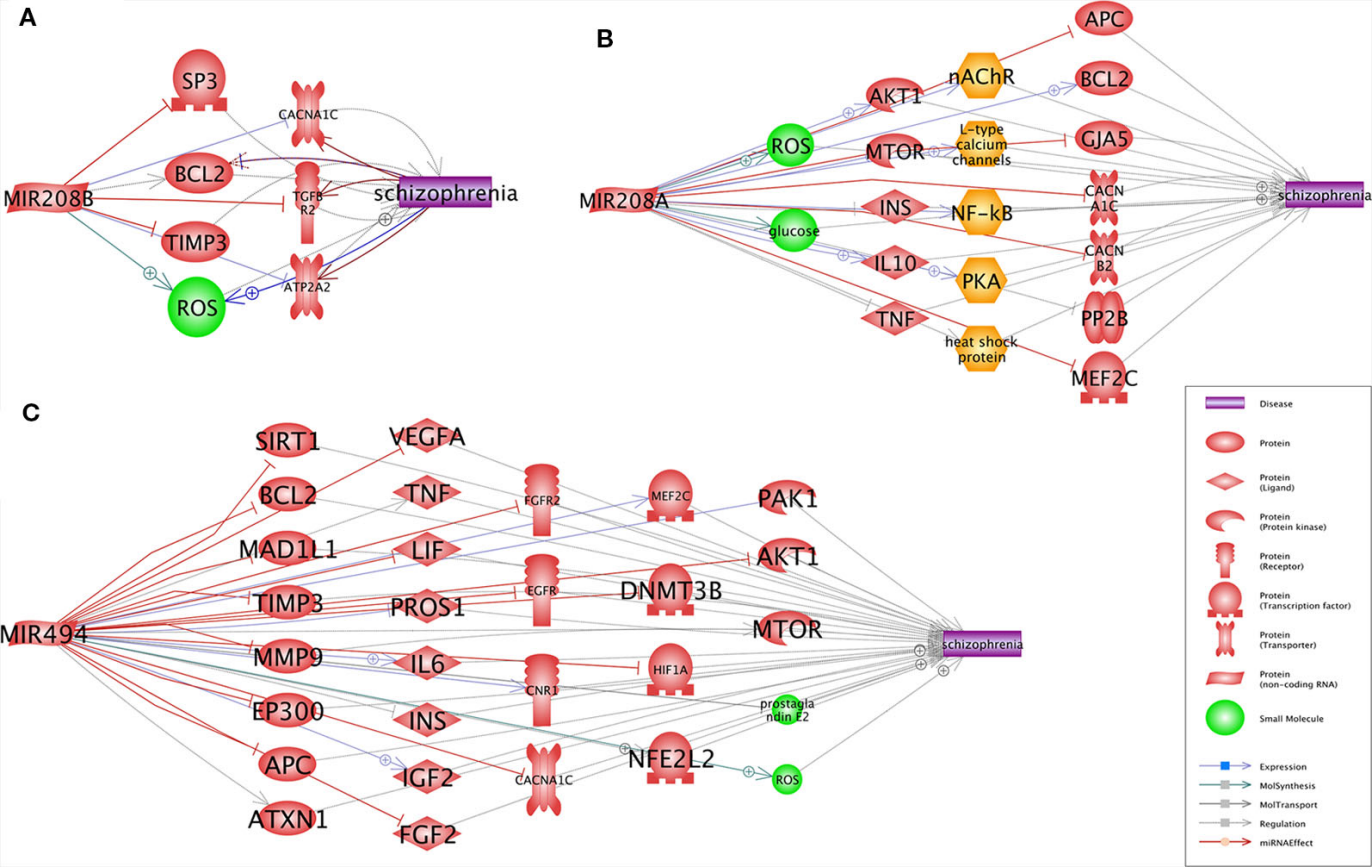
Pathways that link each of three miRNAs, miR-208b **(A)**, miR-208a **(B)**, and miR-494 **(C)**, to schizophrenia. Presented pathways were generated using Pathway Studio (www.pathwaystudio.com) based on known relations mined from existing literature. Each relation has been supported by one or more references summarized in **Supplementary Table 3**.

A Pathway Studio (www.pathwaystudio.com) analysis provided evidence for multiple functional pathways that link miR-208b-3p (**Figure 1A**), miR-208a-3p (**Figure 1B**), and miR-494-5p (**Figure 1C**) to schizophrenia. The relation types and the reference information are presented in **Supplementary Table S3**. Notably, all three schizophrenia-implicated networks regulated by miRNA included BCL2, a well-known regulator of apoptosis and mitochondrial dynamics, and a calcium voltage-gated channel subunit alpha1 C (CACNA1C), one of the L-type calcium channels (LTCCs) defining the calcium influx into cells, and these are critical for normal brain development and plasticity (**Figure 1**).

### Protein–Protein Interaction Among Target Genes of the Three miRNAs

**Figure 2:**
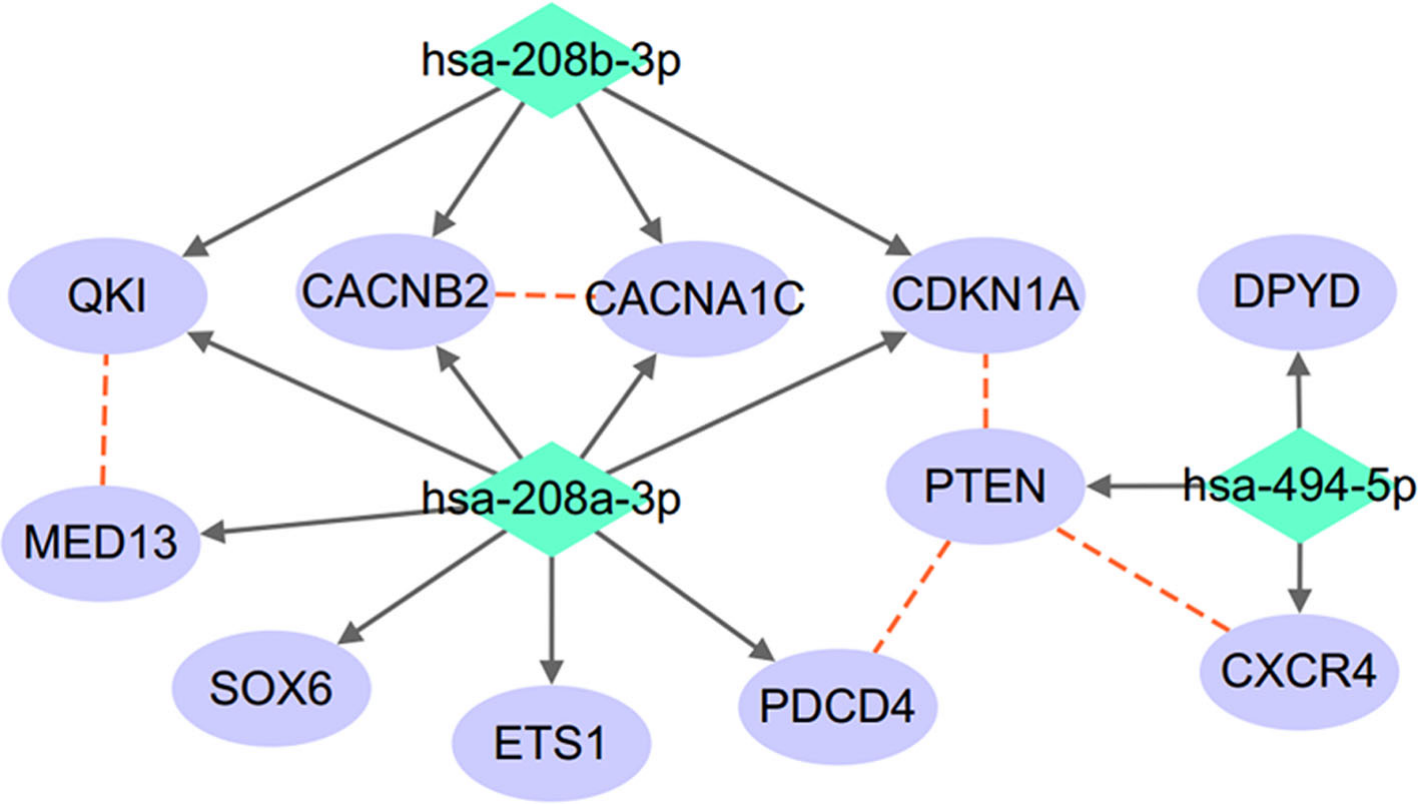
miRNA–target regulatory network connecting miR-208a-3p, miR-208b-3p, and miR-494-5p. Red dashed lines denote protein–protein interactions; solid arrowed lines denote miRNA–target bindings.

A protein–protein interactions (PPIs) analysis was conducted to study the relationship between the target genes of the three miRNAs (miR-208a, miR-208b, and miR-494), as shown in **Figure 2**. The relation data shown in **Figure 2** were acquired from STRING v10.0 (Szklarczyk et al., 2017) and plotted using Cytoscape (Shannon et al., 2003).

As shown in **Figure 2**, the three miRNAs connect to each other through a complex but relatively compact network through multiple common target-binding proteins. Moreover, many protein components of this network are known to interact with each other, suggesting that this network is not random.

### Validation by Association With the Response to the Treatment With Antipsychotic Drugs

To validate the association of miR-208a-3p, miR-208b-3p, and miR-494-5p and their target sets with schizophrenia, a GWAS of antipsychotic treatment response in 2,413 psychiatric patients (Yu et al., 2018) was mined to detecting enrichment. As shown in **Table 3**, the gene set regulated by miR-494-5p was associated with the drug treatment response of patients with schizophrenia.

**Table 3:** miRNA–target gene set analyses with schizophrenia in validation dataset.

miRNA	nGenes	Beta	S.E.	*p*
hsa-miR-494-5p	3	0.971	0.506	0.030
hsa-miR-208a-3p	8	0.097	0.349	0.391
hsa-miR-208b-3p	4	-0.133	0.48	0.608

nGenes: the number of target genes for the miRNA; Beta, the regression coefficient for target gene set analysis; S.E., the standard error of the regression coefficient.

## Discussion

Accumulating evidence suggests that post-transcriptional gene expression regulators, known as microRNAs (miRNAs), play a crucial role in many physiological and pathophysiological processes in human brain. In particular, various areas of the brain and the serum of individuals with schizophrenia were studied for the cellular and extracellular content of miRNA molecules as well as the widespread alterations of their levels reported (Moreau et al., 2011; Santarelli et al., 2011; Banigan et al., 2013). In their typical biomarker discovery design, these and other studies have not aimed at differentiating causal or consequential relationships between the change in the levels of certain miRNA and the development of psychiatric conditions. Nevertheless, the miRNAs encoded by these genes, for example, miR-137 (Kuswanto et al., 2015), were found to harbor the single nucleotide polymorphisms (SNPs) for an increased risk of schizophrenia.

This study highlights three additional miRNAs, hsa-miR-208b-3p, hsa-miR-494-5p, and hsa-miR-208a-3p, as potential contributors to schizophrenia and as the master regulators for the genes previously implicated in this disorder. An analysis of their gene expression showed that these miRNA species were expressed in the brain tissue, the plasma/serum/placenta, or in a combination of these at relatively high levels. Current evidence suggests that the blood–brain barrier does not block the passage of miRNAs between CSF and blood, even if brain-derived miRNAs are somewhat more diluted in blood (Stoicea et al., 2016). While the data on the penetration of miRNA from peripheral tissues to the brain are limited, one can assume that this transfer is highly possible, especially during embryonic development when brain tissue and its compartmentalization is not yet fully formed. Moreover, recent experiments performed in two different rodent models has shown that, in certain conditions, such as during hypoxia, miRNAs actively contribute to an increase in the penetrability of the blood–brain barrier through the inhibition of genes encoding tight junction proteins (Ma et al., 2017; Burek et al., 2019). The role of prenatal and perinatal factors contributing to the risk of schizophrenia was well documented (Kelly and Murray, 2000; Davis et al., 2016). Thereby, one may surmise that the molecular underpinning of this connection may be dependent on plasma miRNAs being carried to the brain in the course of hypoxia or other types of fetal stress, and it may also possibly be dependent on the pathophysiological pairing between miRNAs and mRNAs in non-target tissue.

The accurate identification of miRNA targets remains a formidable challenge. As the output generated by commonly used microRNA–mRNA interaction-predicting software fails to pinpoint experimentally confirmed microRNA-binding regions correctly (Willgoose, 1981; Plotnikova and Skoblov, 2018), we had resorted to limiting our study by investigating only experimentally validated microRNA–mRNA interactions with a subsequent anchoring of them to schizophrenia-related targets by leveraging the data generated over the course of the largest schizophrenia-dissecting GWAS performed to date. Further support for our findings was obtained by the Pathway Studio guided analysis, which allowed us to perform a systems analysis of the molecular pathways engaged by these miRNAs.

Two functional molecules, BCL2 and CACNA1C, were commonly shared between all three miRNA-coordinated “Shortest Path” networks. Notably, both of these molecules were implicated in schizophrenia in numerous previous studies. *CACNA1C*, which encodes for the Ca_v_1.2 α1 subunit of L-type calcium channels (LTCCs), is one of the best-supported risk loci for schizophrenia and bipolar disorder since it harbors variants with consequences on neural processing and connectivity (Gurung and Prata, 2015; Kabir et al., 2017). For BCL2, the connections to schizophrenia are at the level of cellular processes rather than genetic ones. In the astroglia and the neurons, BCL2 regulates autophagy, which maintains the balance between the synthesis, degradation, and recycling of mitochondria and other cellular components (Aouacheria et al., 2017) as well as prevents apoptosis (Almeida, 2013). The networks we built for schizophrenia risk miRNAs imply the disease-associated deregulation of BCL2/BAX and the resultant enhancement in cell susceptibility to apoptosis, which possibly involves an increase in the production of reactive oxygen species (Wu et al., 2013).

If increases in respective miRNA signals are defined genetically, their observed effects should be systemic rather than brain specific. In this light, it is important to note that the primary fibroblasts collected form antipsychotic-naïve patients with first-episode schizophrenia have greater apoptotic susceptibility, higher caspase-3 activity, and lower BCL2 expression than healthy controls (Gassó et al., 2014). Increased expression of hsa-miR-208b-3p, hsa-miR-494-5p, and hsa-miR-208a-3p may augment susceptibility to schizophrenia by simultaneously conferring susceptibility to apoptosis and altering neural processing and connectivity through the suppression of *BCL2* and *CACNA1C*, respectively.

Importantly, all three schizophrenia-contributing miRNA molecules are far from novel. Cardiomyocyte molecules miR-208a-3p and miR-208b-3p belong to the miR-208 family, which participates in ventricular remodeling (Liu et al., 2016) by promoting myocardial fibrosis (Shyu et al., 2015) and apoptosis of cardiomyocytes (Shannon et al., 2003; Luo et al., 2004; Moreau et al., 2011; Tsai et al., 2013; Huang et al., 2016). Both miR-208a-3p and miR-208b-3p reduce the expression of the RNA-binding protein Quaking, encoded by gene *QKI*, which inhibits the apoptosis of cardiomyocytes under ischemia/reperfusion condition (de Bruin et al., 2017; Wang F. et al., 2017). Peculiarly, the dysmyelinating mouse mutant shaking (shk), a model of schizophrenia, is a quaking (qk) allele consisting of a 105-nucleotide insertion in the qk regulatory region that decreases the transcription of qk (Chaverneff et al., 2015). Downregulation of the QKI gene was also noted in the brains of schizophrenic patients (Haroutunian et al., 2006). It was hypothesized that deregulation of QKI underlines the defects of oligodendrocyte differentiation and in myelination detected in schizophrenia (Rosenbluth and Bobrowski-Khoury, 2013) as well as in—as described in a separate study—at least some cases of intellectual disability (Darbelli and Richard, 2016). Moreover, in yet another model tissue, auditory nerves, function of both QKI and its protein product substantially decreases in response to noise exposure, leading to demyelination and hearing deficiency (Panganiban et al., 2018). When QKI-regulating molecules of the miR-208 family are overexpressed, their effects are similar to the decrease in the transcription of QKI and should promote the development of the myelination defects. Remarkably, at clinically relevant concentrations of Haloperidol, the expression levels of QKI-encoding mRNA may be restored (Jiang et al., 2009), which would, in turn, alleviate demyelination-related symptoms.

There is no doubt that miR-208-regulated QKI defines the phenotypic plasticity of the vascular smooth muscle cells (van der Veer et al., 2013; Cochrane et al., 2017). These functional pieces of evidence of the involvement of *QKI* into the development of cardiovascular conditions are also supported by the GWAS, which pointed at *QKI* as a contributor to coronary heart disease (Dehghan et al., 2016). Patients with schizophrenia are known to have higher mortality rates for all major cardiovascular diagnoses (Wu et al., 2015; Westman et al., 2018). It is tempting to speculate that the connection between these two major disabilities may be, at least in part, explained by the sharing of regulatory networks, particularly ones connecting miRNAs of the miR-208 family and *QKI*.

Another pathophysiological process characterized by alterations in *QKI* is the development of gliomas. This gene serves as a tumor suppressor that promotes endolysosome-mediated degradation and suppresses the display of receptors essential for maintaining the self-renewal of neural stem cells outside their niche (Shingu et al., 2017). Consequently, the *QKI* gene tends to be eliminated in gliomas, either through a complete deletion or through a disruption by translocation (Bandopadhayay et al., 2016). While the roles for upstream regulators of *QKI*, hsa-miR-208b-3p and hsa-miR-208a-3p, in glioma have not yet been described, another miRNA that affects schizophrenia risk, hsa-miR-494-5p, is a definite glioma suppressor (Li et al., 2015; Zhang et al., 2015; Xu et al., 2018). Importantly, in the case of the latter miRNA, protection against the development of the tumors comes at a cost of elevated susceptibility to neurotoxicity, after exposure to ischemia/reperfusion for example. Notably, knockdown of hsa-miR-494-5p reverses the neurotoxic phenotype in multiple models (Song et al., 2017; Deng et al., 2019; Zhao et al., 2019a; Zhao et al., 2019b). Hsa-miR-494-5p-dependent antagonistic relationships between gliomagenesis and neurotoxicity are intriguing, as they support previously noted decrease in odds of the development of brain tumors in patients with schizophrenia (Grinshpoon et al., 2005; Levav et al., 2007; Wang Y. et al., 2017).

## Conclusion

In summary, the presented results point at an orchestrating role of miRNAs in the pathophysiology of schizophrenia. Cellular effects of risk-associated miRNAs, namely, hsa-miR-208b-3p, hsa-miR-494-5p, and hsa-miR-208a-3p, align with the primary etiological hypotheses of schizophrenia and suggest that the three molecules, as well as their target genes, should be investigated for possible pharmacological interventions. The sharing of regulatory networks between schizophrenia and other pathologies may explain higher cardiovascular mortality and lower odds of glioma previously reported in psychiatric patients. Molecular tools for manipulating miRNA activity, including miRNA sponges, are already being developed for cancers (Jung et al., 2015; Fang et al., 2017) and for cardiovascular disease (Bernardo et al., 2018). There is a hope that similarly designed therapeutic interventions may find their utility in the treatment of schizophrenia and other life-long psychiatric illnesses.

## Data Availability Statement

The datasets generated for this study can be found in the **Supplementary Material**.

## Author Contributions

HC, DL, and FZ developed the study design. FZ, WY, HY, ZZ, HC, and AB analyzed the data. FZ, HC, and AB drafted and then edited the original paper. All authors read and approved the final manuscript.

## Funding

The study was supported the National Natural Science Foundation of China (No. 81471364) and Primary Research & Development Plan of Jiangsu Province (BE2016630).

## Conflict of Interest

Author HC was employed by the company Elsevier Inc.

The remaining authors declare that the research was conducted in the absence of any commercial or financial relationships that could be construed as a potential conflict of interest.

## Supplementary Material

Supplementary Table S1 miRNA-target gene pairs retrieved from MiRTarBase.

Supplementary Table S2 Enrichment of miRNA Target Set in PGC2.

Supplementary Table S3 References supporting the literature-based pathway linking SCZ and the three miRNAs.
